# Mouse taste cells with G protein-coupled taste receptors lack voltage-gated calcium channels and SNAP-25

**DOI:** 10.1186/1741-7007-4-7

**Published:** 2006-03-30

**Authors:** Tod R Clapp, Kathryn F Medler, Sami Damak, Robert F Margolskee, Sue C Kinnamon

**Affiliations:** 1 Department of Biomedical Sciences, Colorado State University, Fort Collins, CO 80523, USA; 2 Rocky Mountain Taste and Smell Center, UCDHSC, Aurora, CO, USA; 3 Department of Biological Sciences, University at Buffalo, Buffalo, NY, USA; 4 Dept. of Physiology and Biophysics, Mount. Sinai School of Medicine, New York, NY, USA; 5 Department of Neuroscience, Mount Sinai School of Medicine, New York, NY, USA; 6 SD, Nestle Research Center, Vers-chez-les-Blanc, Lausanne, Switzerland

## Abstract

**Background:**

Taste receptor cells are responsible for transducing chemical stimuli from the environment and relaying information to the nervous system. Bitter, sweet and umami stimuli utilize G-protein coupled receptors which activate the phospholipase C (PLC) signaling pathway in Type II taste cells. However, it is not known how these cells communicate with the nervous system. Previous studies have shown that the subset of taste cells that expresses the T2R bitter receptors lack voltage-gated Ca^2+ ^channels, which are normally required for synaptic transmission at conventional synapses. Here we use two lines of transgenic mice expressing green fluorescent protein (GFP) from two taste-specific promoters to examine Ca^2+ ^signaling in subsets of Type II cells: T1R3-GFP mice were used to identify sweet- and umami-sensitive taste cells, while TRPM5-GFP mice were used to identify all cells that utilize the PLC signaling pathway for transduction. Voltage-gated Ca^2+ ^currents were assessed with Ca^2+ ^imaging and whole cell recording, while immunocytochemistry was used to detect expression of SNAP-25, a presynaptic SNARE protein that is associated with conventional synapses in taste cells.

**Results:**

Depolarization with high K^+ ^resulted in an increase in intracellular Ca^2+ ^in a small subset of non-GFP labeled cells of both transgenic mouse lines. In contrast, no depolarization-evoked Ca^2+ ^responses were observed in GFP-expressing taste cells of either genotype, but GFP-labeled cells responded to the PLC activator *m*-3M3FBS, suggesting that these cells were viable. Whole cell recording indicated that the GFP-labeled cells of both genotypes had small voltage-dependent Na^+ ^and K^+ ^currents, but no evidence of Ca^2+ ^currents. A subset of non-GFP labeled taste cells exhibited large voltage-dependent Na^+ ^and K^+ ^currents and a high threshold voltage-gated Ca^2+ ^current. Immunocytochemistry indicated that SNAP-25 was expressed in a separate population of taste cells from those expressing T1R3 or TRPM5. These data indicate that G protein-coupled taste receptors and conventional synaptic signaling mechanisms are expressed in separate populations of taste cells.

**Conclusion:**

The taste receptor cells responsible for the transduction of bitter, sweet, and umami stimuli are unlikely to communicate with nerve fibers by using conventional chemical synapses.

## Background

Taste buds, the transducing elements of gustatory sensation, contain a heterogeneous population of 50 to 100 elongate taste receptor cells, which extend from the basal lamina to the surface of the epithelium. Taste stimuli interact with receptors on the apical membrane, while the basolateral membranes of some taste cells associate with gustatory nerve fibers to transmit taste information to the brain.

Several types of taste cells have been identified morphologically. Type I cells, also known as "dark" cells, generally comprise about half of the taste bud. These cells are not believed to have a receptive function, but to play a more glial-like role in the taste bud [1,2]. About 35% of the cells are Type II cells, which are also known as "light" cells due to the electron lucent nature of their cytoplasm. Type II cells express T1R and T2R taste receptor proteins and downstream signaling effectors for bitter, sweet, and umami taste stimuli [3-5]. These cells associate with afferent nerve fibers, but do not form morphologically identifiable synapses [4]. The remaining cells are Type III cells, sometimes referred to as "intermediate" cells. Most, if not all of these cells lack receptors and signaling effectors for bitter, sweet, and umami stimuli but may be involved in sour taste transduction [6]. Unlike Type II cells, Type III cells form synapses with nerve fibers that contain prominent pre and post synaptic specializations [7-9]. These observations have raised questions about the mechanisms used by Type II cells to communicate bitter, sweet, and umami information to the nervous system.

We have begun to address this question by characterizing the membrane properties and synaptic proteins expressed by Type II cells in mouse circumvallate taste buds. Type II cells are divisible into at least two subsets: one contains the T2R family of bitter receptors, while the other contains the T1R family of taste receptors, which normally exist as heterodimers. T1R1 + T1R3 is a broadly tuned amino acid receptor [10], while T1R2 + T1R3 forms a receptor responsive to most sweet stimuli [11,12]. Both T2Rs and T1Rs activate the PLC signaling cascade, eliciting release of Ca^2+ ^from intracellular stores and presumed activation of the monovalent-selective cation channel, TRPM5 [13-15]. Recent electrophysiological studies suggest that the bitter-sensitive, α-gustducin-expressing subset of Type II cells lack voltage-gated Ca^2+ ^channels and have only small voltage-gated Na^+ ^and K^+ ^currents [16]. Do the T1R3-expressing Type II cells also lack voltage-gated Ca^2+ ^channels? These cells are of particular interest, because sweet stimuli generate large trains of action potentials, which might be expected to activate voltage-gated Ca^2+ ^channels [17-19]. To address this question, we used electrophysiology and Ca^2+ ^imaging of taste cells isolated from transgenic mice expressing GFP from either the T1R3 promoter (which labels sweet and/or umami-sensitive taste cells) or the TrpM5 promoter (which labels the entire subset of PLC expressing cells) to ask whether any taste cells co-express PLC signaling components and voltage-gated Ca^2+ ^channels. In addition, we used immunoctyochemistry to determine if these taste cells express the target SNARE protein, SNAP-25, which has previously been associated with synapses in taste cells [20]. Our data suggest that the entire subset of taste cells that use the PLC signaling pathway for transduction lack conventional synapses with the nervous system.

## Results

### Identification of receptor-expressing Type II taste cells

**Figure 1:**
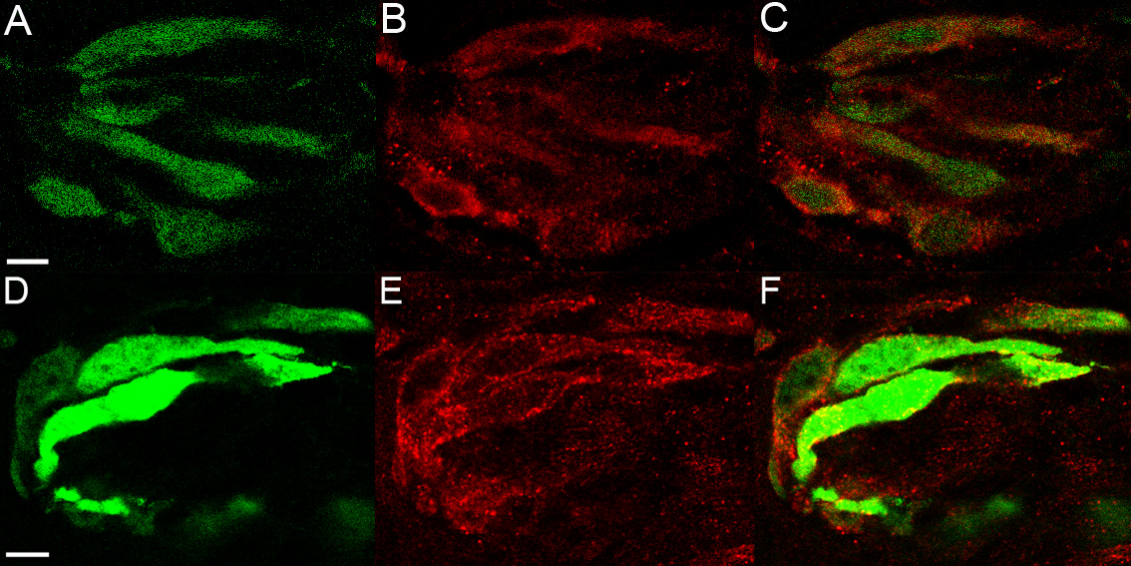
Laser scanning confocal micrographs (LCSMs) of mouse circumvallate taste buds show that GFP is an accurate reporter of protein. Panels A-C show sections from transgenic mice expressing GFP under the control of the T1R3 promoter. Green denotes T1R3 GFP expression (A, C) and red shows T1R3-like-immunoreactivity (LIR) (B, C). Panels D-F show sections taken from mice expressing GFP under control of the TRPM5 promoter. Green denotes TRPM5 GFP expression (D, F) and red shows TRPM5-LIR (E, F). Scale bar is 20 μm.

Immunocytochemistry was performed to demonstrate that the GFP expression is representative of protein expression. We compared T1R3-like immunoreactivity (LIR) to T1R3-GFP and TRPM5-LIR to TRPM5-GFP. Most T1R3-LIR and TRPM5-LIR was apparent in the membrane region and absent from the nuclear region. These immunoreactive cells have large round nuclei typical of Type II taste cells. Immunoreactivity was confined to taste cells and not observed in the epithelium surrounding taste buds or nerve fibers. GFP differed from immunoreactivity in that it was seen throughout the cytosol and in the nuclear region. Cells were counted as being GFP positive and/or immunoreactive only if a nuclear region and apical extension could be seen. In the T1R3-GFP animals we counted 41 taste buds and 95 cells from 4 mice. All T1R3-LIR cells expressed GFP and 82% of the T1R3-GFP positive cells displayed T1R3-LIR (Figure 1A). The high background observed when using the T1R3 antibody may account for the small difference between T1R3-GFP and T1R3-LIR. In 49 taste buds from 4 mice, 200 TRPM5-GFP positive cells all displayed TRPM5-LIR and vice versa (Figure 1B). We conclude that T1R3-GFP and TRPM5-GFP are representative of the population of cells expressing these proteins. Control sections in which the primary antibody was omitted showed no labeling.

### Ca^2+ ^responses in T1R3-GFP cells

**Figure 2:**
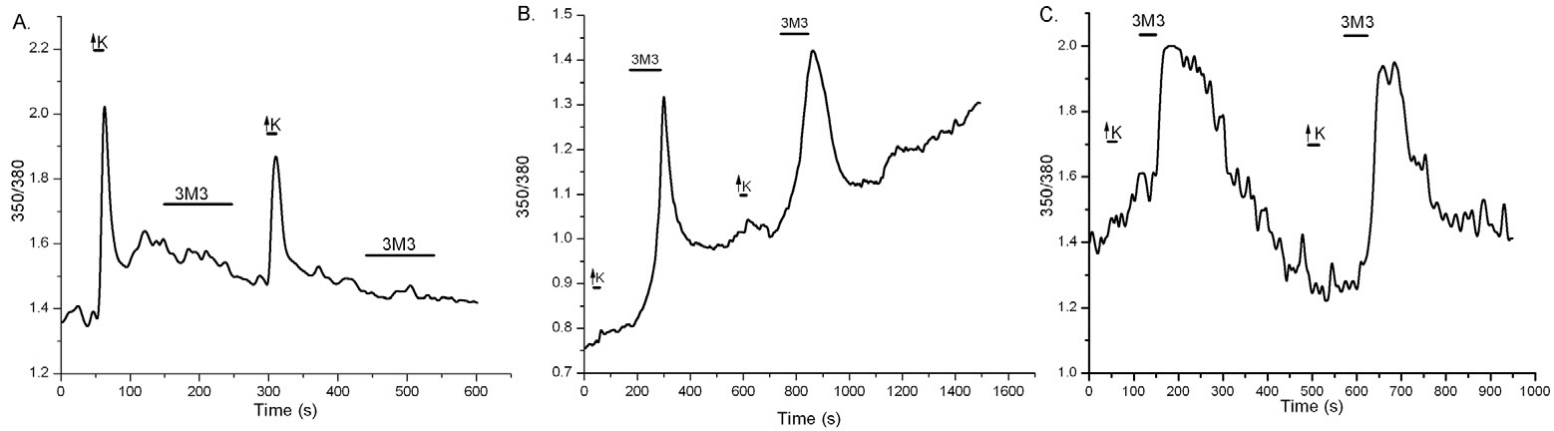
A depolarizing stimulus of high K^+ ^solution does not increase [Ca^2+^]_i _in bitter, sweet, and umami sensitive cells. Panel A shows an isolated non-GFP cell from a T1R3-GFP expressing animal. Some non-GFP cells do present an increase in [Ca^2+^]_i _when challenged with high K^+ ^solution. Cells that have [Ca^2+^]_i _responses to high K^+ ^do not respond to the PLC agonist *m*-3M3FBS, 10 μM. T1R3-GFP expressing cells in Panel B do not show changes in [Ca^2+^]_i _to bath applied high K^+ ^but do show reversible increases in [Ca^2+^]_i _to bath applied PLC agonist *m*-3M3FBS (10 μM). TRPM5-GFP expressing cells in Panel C do not change [Ca^2+^]_i _to high K^+ ^but show reversible increases in [Ca^2+^]_i _when 10 μM *m*-3M3FBS is present.

Voltage gated Ca^2+ ^channels (VGCCs) are usually associated with conventional synapses. In neurons, these channels are responsible for the depolarization-induced Ca^2+ ^influx required for vesicular release. Sweet responsive taste cells are known to generate trains of action potentials [17-19], suggesting they may express voltage-gated Ca^2+ ^channels. To determine if T1R3-expressing taste cells possess VGCCs, we loaded taste cells isolated from T1R3-GFP mice with bath-applied fura2-AM, identified cells as GFP^+ ^or GFP^-^, and used ratiometric imaging to monitor Ca^2+ ^responses. The field of view often contained both GFP-expressing and non-GFP-expressing cells. Cells were challenged with a depolarizing high K^+ ^solution followed by wash in Tyrode's. No T1R3-GFP cells responded to the high K^+ ^depolarizing stimulus (n = 21), however, fourteen non-GFP cells showed a sharp Ca^2+ ^increase in response to high K^+ ^(Figure 2A). These responses were repeatable with little run down, and demonstrate that the cell preparation contained cells with viable VGCCs. As an additional control for cell viability, we examined Ca^2+ ^responses to the PLC activator, *m*-3M3FBS. We expected all GFP-expressing cells to exhibit a Ca^2+ ^response to the activator, because T1R3 couples to the PLC signaling pathway [13]. Most T1R3-GFP cells and few non-GFP cells showed a Ca^2+ ^response to 10 μM *m*-3M3FBS (Figure 2B). Responses to the PLC activator were blocked by the PLC inhibitor, U73122, indicating that *m*-3M3FBS is activating PLC in taste cells (data not shown). Responses to the PLC activator varied among cells in that some exhibited a return to baseline while others did not, effectively killing the cell. GFP expressing taste cells that did not show a Ca^2+ ^response to *m*-3M3FBS were eliminated from further analysis.

### Ca^2+ ^responses in TRPM5-GFP cells

TRPM5 is co-expressed with other PLC signaling effectors found in Type II cells [4,21]. These components are downstream of the T2R and T1R taste receptors for bitter, sweet, and umami taste ([13]). To examine this larger population of cells, we isolated taste cells from TRPM5-GFP expressing mice. Cells were loaded with bath applied fura2-AM and assessed as GFP^+ ^or GFP^-^. All TRPM5-GFP expressing taste cells tested (n = 20) failed to show increases in Ca^2+ ^in response to high K^+ ^depolarization, although they showed Ca^2+ ^responses to the PLC activator 3M3FBS (Figure 2C). Similar to the T1R3-GFP mice, a small subset of non-GFP expressing taste cells showed sharp and repeatable increases in intracellular Ca^2+ ^to high K^+^, suggesting that the cell population was viable.

### Whole cell voltage clamp recording

**Figure 3:**
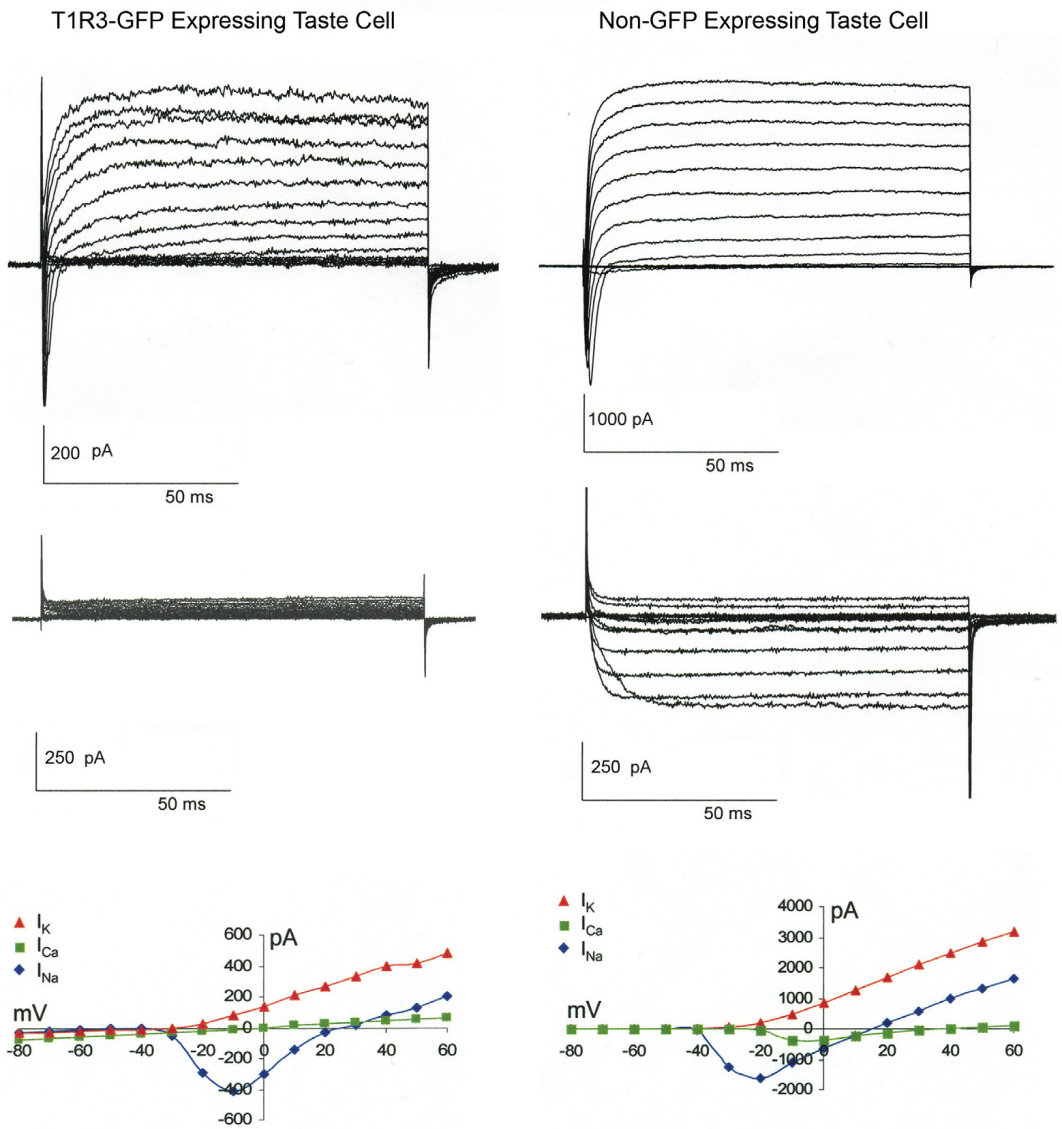
Whole cell voltage clamp recordings from a T1R3-GFP expressing taste cell (left panel) and a non-GFP-expressing taste cell (right panel). Holding potential was -80 mV, and the membrane was stepped from -80 to +60 mV to elicit voltage-gated currents. Top panels show recordings in Tyrode's, while middle panels show recordings in Ba^2+ ^Tyrode's to reveal Ba^2+ ^currents through voltage gated Ca^2+ ^channels. Bottom panels illustrate the current/voltage relationship.

Since many VGCCs show at least some inactivation with prolonged depolarization, it is possible that we failed to see Ca^2+ ^responses in some cells due to damage during isolation or photo bleaching of GFP during Ca^2+ ^imaging. To control for this possibility, we used whole cell voltage-clamp recording of T1R3-GFP labeled taste cells as an additional assay for the presence of VGCCs. In normal Tyrode's solution, all taste cells tested (n = 9) exhibited small voltage-gated Na^+ ^and K^+ ^currents in response to 10 mV depolarizing steps from a holding potential of -80 mV. To test for the presence of VGCCs, we treated cells with a Tyrode's solution containing BaCl_2_, TTX, and TEA. (c.f., [16]). Under these conditions, no inward currents were elicited, suggesting that these cells do not possess VGCCs (Figure 3). As a control for the methodology, we also tested several non-GFP expressing taste cells. A small subset of these cells displayed large voltage-gated Na^+ ^and K^+ ^currents and a large, slowly inactivating inward current in the BaCl_2 _Tyrode's (Figure 3). The I/V profile of this current suggests it is elicited by high threshold VGCCs, possibly of the L or P/Q type (Medler, unpub. observations). These whole cell data corroborate our Ca^2+ ^imaging data and suggest that the lack of response to high K^+ ^depolarization in GFP positive cells is not due to inactivation of voltage-gated Ca^2+ ^channels.

### T1R3 and SNAP-25 expression

**Figure 4:**
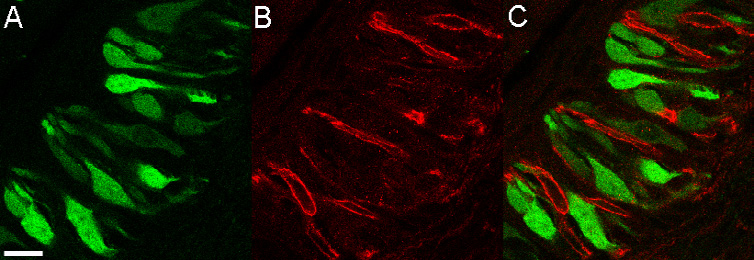
LCSMs from mouse circumvallate papillae showing T1R3 promoter driven GFP expression and SNAP-25-LIR. Green denotes GFP expression (A, C) and red SNAP-25-LIR (B, C). The GFP+ cells appear to be a separate population than those with SNAP-25-LIR. Scale bar is 20 μm.

The presynaptic protein SNAP-25 is part of the SNARE complex that plays a role in the release of neurotransmitter from cells (for a review see [22]). Immuno electron microscopy studies on rat taste buds showed that SNAP-25 is expressed in appromiately 98% of taste cells with conventional synapses [20]. To determine if T1R3-expressing taste cells display evidence of SNAP-25 expression, we used immunocytochemistry to identify mouse taste cells expressing SNAP-25 and compared this to the expression of T1R3-GFP. We examined 32 40 μm sections from circumvallate taste buds of four mice and found no overlap between SNAP-25-LIR and T1R3-GFP expression (Figure 4).

### TRPM5 and SNAP-25 expression

**Figure 5:**
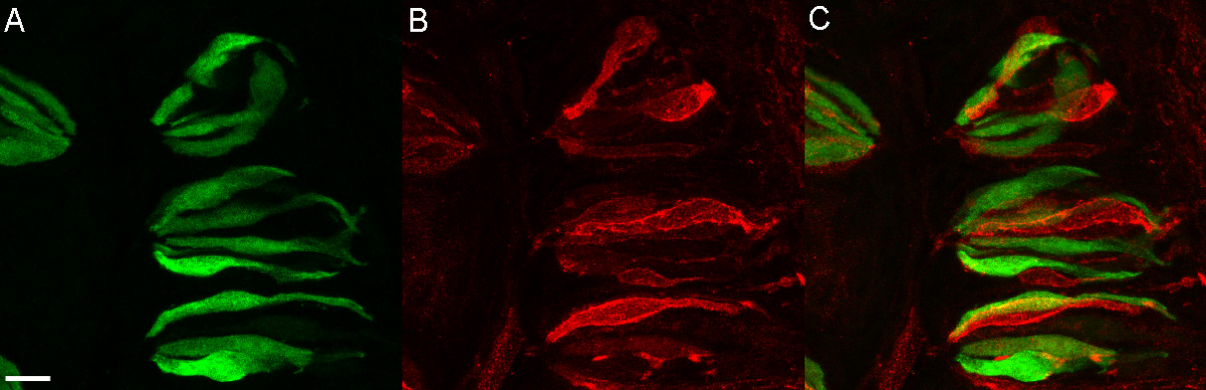
Z-series stack of five LCSMs 0.5 μm apart from mouse circumvallate papillae showing TRPM5 promoter driven GFP expression and SNAP-25-LIR. Green denotes GFP expression (A, C) and red SNAP-25-LIR (B, C). The GFP+ cells appear to be a separate population than those with SNAP-25-LIR. Scale bar is 20 μm.

T1R3 is present in a subset of the cells that express TRPM5, which is believed to be expressed in all Type II taste cells. To determine if the entire TRPM5 population lacks expression of SNAP-25, we tested circumvallate papillae from the TRPM5-GFP mice with the antibody to SNAP-25. We examined 32 sections from four mice, but failed to find any overlap between TRPM5 expression and SNAP-25-LIR (Figure 5).

## Discussion

Our data using multiple approaches strongly suggest that the taste receptor cells responsible for sweet, umami, and bitter taste transduction lack voltage-gated Ca^2+ ^channels and presynaptic machinery normally required for conventional synaptic transmission. These data are consistent with other physiological [16], molecular [23], and ultrastructural [4] findings suggesting that PLC expressing taste cells in rodents lack conventional synapses with the nervous system. However, Medler et al. [16] reported that one subset of Type II cells had voltage-gated Ca^2+ ^channels. Type II cells in that study were identified by immunoreactivity to an external epitope marker, Antigen A, that was reported to be expressed selectively on Type II taste cells in rats [24]. Antigen A immunoreactivity has not been examined at the ultrastructural level in mouse taste buds, so it is possible that Antigen A is labeling some Type III cells in addition to Type II cells. Alternatively, it is possible that some Type II cells in mouse do not express PLC signaling components. Further studies will be required to resolve this discrepancy. Our data also differ significantly from those of Oike et al. [25], who recently reported a large number of PLCβ2-expressing cells also express SNAP-25-LIR. Their studies involved rat taste buds, so it is possible that a species difference is the source of the discrepancy. We do not believe this to be the case, since ultrastructural studies show that PLCβ2 does not appear to be expressed in rat taste cells with conventional synapses [4], which Yang et al. [20] earlier showed express SNAP-25-LIR.

The lack of voltage-gated Ca^2+ ^channels is surprising, because Type II taste cells have voltage-gated Na^+ ^and K^+ ^channels (c.f,., Figure 3) and generate action potentials to at least sweet stimuli [17-19]. In addition, TRPM5 is a monovalent cation channel and thus is unable to contribute to the Ca^2+ ^influx needed for synaptic transmission [15,26]. TRPM5 is, however, clearly critical for bitter, sweet, and umami transduction [13,27], suggesting that it contributes to membrane depolarization, which apparently is required for transduction and afferent signaling. How is taste information from Type II receptor cells communicated to afferent nerve fibers? One possibility is that Type II cells communicate with the nervous system through the agency of Type III cells. Such communication may involve gap junctions, which were recently shown to be present in mouse taste buds [28]. Action potentials elicited by taste stimuli in Type II cells would depolarize Type III cells, which would activate afferent nerve fibers by conventional synaptic mechanisms. Ultrastructural studies have shown that Type III cells form conventional synapses with the nervous system [29,30,20,9]. Further, electrophysiological studies revealed that Type III cells, identified by the Type III cell marker NCAM, have VGCCs [16]. Thus, Type III cells have the properties necessary to serve as output cells for the taste bud.

An alternate hypothesis is that Type II cells communicate directly with afferent nerve fibers, but use unconventional mechanisms. Afferent nerve fibers do associate with Type II taste cells and form subsurface cisternae at sites of contact, although it is not clear how these specializations might contribute to afferent signaling [4]. Type II taste cells contain vesicles and the vesicle SNARE protein synaptobrevin-2 [31], although the vesicles are not usually associated with nerve fiber contact. It has recently been shown that the purinergic receptors P2X2 and P2X3, expressed on gustatory afferent nerve fibers, are required for transmission of taste information to the nervous system [32]. Perhaps ATP is released via a non-conventional mechanism from PLC signaling cells to activate nerve fibers directly. In neurons, ATP release is usually vesicular and Ca^2+ ^dependent [33]. However, in non-neuronal cells, ATP can be released by a number of unconventional mechanisms, including voltage-dependent anion channels and gap junction hemi channels [34,35]. Further studies will be required to determine if Type II taste cells communicate directly with nerve fibers or through the agency of Type III cells.

## Conclusion

The principal finding in this study is that the cells responsible for bitter, sweet, and umami taste transduction do not have conventional synapses with the nervous system. Two separate lines of transgenic mice allowed identification of the sweet- and umami-sensitive cells, T1R3-GFP, and the larger subset of bitter-, sweet-, and umami-sensitive cells, TRPM5-GFP. Both subsets, T1R3-GFP and TRPM5-GFP, lack VGCCs. Further, they lack the target SNARE protein SNAP-25 that has previously been associated with taste cells that have morphologically identifiable synapses [20]. We conclude that PLC expressing taste cells do not communicate with the nervous system via conventional synaptic mechanisms.

## Methods

### Animals

Adult transgenic mice in which either the T1R3 or TRPM5 promoter drives expression of GFP were used. Animals were cared for in compliance with the Colorado State University Animal Care and Use Committee. Specifically, pIRES2-eGFP containing the encephalomyocarditis virus internal ribosome entry site (IRES) and enhanced green fluorescent protein (eGFP) was purchased from Clontech (Palo Alto, CA). The wheat germ agglutinin (WGA) cDNA was a gift from Dr N.V. Raikhel. The T1r3 and Trpm5 genes were subcloned from BACs obtained by screening a C57BL6 mouse BAC library. The construct T1R3-GFP contained 5' to 3': 13 kb of the mouse T1r3 gene including the 5' flanking region and the entire 5' untranslated region, the WGA cDNA, IRES and eGFP. WGA was included in the construct for the purpose of tracing studies that are unrelated to the studies described here. The construct TRPM5-GFP contained 5' to 3': 11 kb of mouse Trpm5 5' flanking sequence, Trpm5 Exon 1 (untranslated), Intron 1, and the untranslated part of Exon 2, and eGFP. The constructs were separated from the vector by restriction endonuclease digestion, purified from agarose gels using a Qiagen kit and microinjected into B6C3 mouse zygotes according to standard methods [36]. Founder transgenic mice were bred to wild-type C57BL6/J mice and their transgenic offspring were used for further experiments.

### Taste cell isolation

Circumvallate and foliate taste buds from TRPM5-GFP and T1R3-GFP mice were isolated using the protocol of Béhé et al. [17]. Briefly, mice were killed with CO_2 _and cervical dislocation. An enzyme cocktail consisting of 1 mg/ml collagenase A or B (Roche, Indianapolis, IN), 3 mg/ml Dispase II (Roche, Indianapolis, IN), and 1 mg/ml trypsin inhibitor (Sigma, St. Louis, MO) dissolved in Tyrode's was injected beneath the epithelium of the tongue. After incubation for 40–45 min. in oxygenated nominally Ca^2+ ^free Tyrode's, the epithelium was gently separated from the underlying connective tissue and placed in Ca^2+ ^free Tyrode's containing 1 mM BAPTA for 10 min. Taste buds were removed by gentle suction with a fire-polished pipet and plated onto cover slips coated with Cell Tak (BD Biosciences, Bedford, MA) or poly-L-lysine (Sigma, St. Louis, MO). This procedure resulted in isolated taste cells, small cell clusters, and entire taste buds. All data for both Ca^2+ ^imaging and whole cell recording were obtained from isolated taste cells or cell clusters so that the presence or absence of GFP expression could be verified.

### Ca^2+ ^imaging

Intracellular Ca^2+ ^measurements were obtained using ~2 μM fura-2 AM (Molecular Probes, Invitrogen Corporation). Images were acquired with the CCD Sensicam QE camera (COOKE Corporation) through a 40× oil immersion objective lens of an inverted Nikon Diaphot TMD microscope. Excitation wavelengths of 350 nm and 380 nm were used and with an emission wavelength ~510 nm. Calcium levels were reported as F350/F380 versus time. Images were captured every 1–5 seconds using Imaging Workbench 5.2 (Indec Biosystems, Inc.) and graphed using OriginPro 7.5 software. Responses were measured to *m*-3M3FBS (PLC activator, 10 μM, Calbiochem, San Diego, CA) and high potassium solution (55 mM). All solutions were bath applied using a gravity flow perfusion system (Automate Scientific Inc., San Francisco, CA) and laminar flow perfusion chambers (RC-25F, Warner Scientific Inc., Hamden, CT).

### Whole cell recording

Voltage dependent currents were measured using the whole cell patch clamp technique [37]. Patch electrodes were pulled from LE-16 glass (Dagan Corporation, MN) with a Flaming/Brown micropipette puller (Model P-97, Sutter Instruments, Novato, CA). Pipette resistance ranged from 2 to 5 MΩ. Seals were obtained using gentle suction and entry into the cell was made further suction, or with a 1 ms depolarizing pulse to the pipette. Whole cell currents were measured using an Axopatch 200A patch clamp amplifier and recorded using pClamp 9 software (Molecular Devices, Sunnyvale, CA). Signals were filtered at 5 kHz and recorded at 100 μs. Membrane capacitance was compensated electronically and external solutions were delivered by gravity flow perfusion (Automate Scientific Inc., San Francisco, CA) at a rate of 2–3 ml/min. Voltage-gated currents were elicited by 100 ms step depolarizations from a holding potential of -80 mV.

### Solutions

#### Ca^2+ ^imaging experiments

Tyrode's solution contained the following (in mM): 140 NaCl, 5 KCl, 1 MgCl_2_, 1 CaCl_2_, 10 HEPES, 10 glucose, and 1 pyruvic acid, adjusted to pH 7.4 with NaOH. High K^+ ^solution contained the following (in mM): 90 NaCl, 55 KCl, 1 MgCl, 1 CaCl_2_, 10 HEPES, 10 glucose, and 1 pyruvic acid. The PLC activator m-3M3FBS was diluted to 10 μM in Tyrode's solution. Ca^2+^/Mg^2+ ^free Tyrode's solution contained 1 mM BAPTA.

#### Patch clamp experiments

Tyrode's was identical to that for Ca^2+ ^imaging, except CaCl_2 _was 2 mM. For recording Ca^2+ ^current, extracellular solution contained (in mM): 136 tetraethylammonium bromide, 10 BaCl_2_, 1 MgCl_2_, 10 HEPES, 10 glucose, 1 pyruvate, and 200 nM TTX to block voltage Na^+ ^currents. Intracellular pipet solution contained (in mM): 140 KCl, 2 MgCl_2_, 1 CaCl_2_, 11 EGTA, 10 HEPES, and 2.5 ATP, adjusted to pH 7.2 with KOH.

### Tissue preparation for immunocytochemistry

Mice were killed in a charged CO_2 _container followed by cervical dislocation. Tongues were removed and immediately placed into fresh 4% paraformaldehyde (Electron Microscopy Sciences, Ft. Washington, PA) in 0.1 M phosphate buffer for approximately two hours. Tongues were then put into a 20% sucrose solution in 0.1 M phosphate buffer overnight for cryoprotection. Forty micron sections were cut on a Leitz 1729 digital Kryostat and collected in 0.1 M phosphate buffered saline (PBS, pH 7.2). Following sectioning, the slices were washed in PBS three times for ten minutes each at room temperature. All sections were incubated in blocking solution for 1–2 hours at room temperature. Blocking solution contained 0.3% Triton X-100, 1% normal goat serum, and 1% bovine serum albumin in 0.1 M PBS. All chemicals were purchased from Sigma Chemical Corporation (St. Louis, MO) unless otherwise noted

### Antibodies

Anti-SNAP-25 polyclonal antibodies raised in rabbit against a 12 residue synthetic peptide (C)ANQRATKMLGSG based on mouse SNAP-25 residues 195–206 were purchased from Calbiochem (San Diego, CA, cat #567343). The secondary Cy-5 anti-rabbit antibody (cat# 111-175-1444) was purchased from Jackson ImmunoResearch Laboratories (West Grove, PA). PA1-740 neutralizing peptide was purchased from Affinity BioReagents (Golden, CO, cat# PEP-032)

### Immunocytochemistry

Sections from mouse circumvallate papillae were incubated with the primary antibody overnight at 4°C. The primary antibody was diluted (1:1000 for anti-TRPM5; 1:500 for anti-T1R3; 1:200 for anti-SNAP-25) in blocking solution. Sections were then washed three times for ten minutes each in PBS at room temperature then incubated with the secondary antibody (1:400) at room temp for two hours. Following incubation with the secondary antibody, sections were washed three times for ten minutes in PBS and mounted on RITE-ON micro slides (Becton, Dickinson and Company, Portsmouth, NH) using Flouromount-G (Southern Biotechnology Associates, Birmingham, AL, cat# 0100-01) and cover slipped (VWR Scientific, Media, PA). Slides were stored at 4°C.

Controls for light microscopy consisted of omitting the primary antiserum and secondary antibody separately. No immunoreactivity was seen under these conditions. We also performed a peptide block for the primary anti-SNAP-25 in which we incubated PA1-740 neutralizing peptide (Affinity Bioreagents, Golden, CO) at equal weight of peptide per volume antibody overnight at 4 degrees C. We found no immunoreactivity following peptide block.

### Confocal microscopy

Images were captured with an Olympus FVX-IHRT Fluoview Confocal Laser Scanning Microscope. Lasers included Argon 488 nm, HeNe 543 nm, and HeNe 622. Fluoview software was used for data acquisition. Sequential scanning techniques were used and showed no differences from simultaneous scans. Images were processed and printed using Adobe Photoshop CS2 software.

## Authors' contributions

The transgenic mouse lines were created by SD and RFM. Calcium imaging and immunocytochemistry were done by TRC. Patch clamp recording were performed by SCK and the study was conceived by KFM and SCK. All authors read and approved the final manuscript.
